# Joint‐linkage mapping and GWAS reveal extensive genetic loci that regulate male inflorescence size in maize

**DOI:** 10.1111/pbi.12519

**Published:** 2016-01-23

**Authors:** Xun Wu, Yongxiang Li, Yunsu Shi, Yanchun Song, Dengfeng Zhang, Chunhui Li, Edward S. Buckler, Yu Li, Zhiwu Zhang, Tianyu Wang

**Affiliations:** ^1^ Institute of Crop Science Chinese Academy of Agricultural Sciences Beijing China; ^2^ Institute for Genomic Diversity Cornell University Ithaca NY USA; ^3^ USA Department of Agriculture‐Agricultural Research Service Ithaca, NY USA; ^4^ Department of Agronomy Northeast Agricultural University Harbin Heilongjiang China; ^5^ Nanchong Academy of Agricultural Sciences Nanchong Sichuan China; ^6^ Department of Crop and Soil Sciences Washington State University Pullman WA USA

**Keywords:** maize, male inflorescence size, joint‐linkage, GWAS, QTLs, candidate gene

## Abstract

Both insufficient and excessive male inflorescence size leads to a reduction in maize yield. Knowledge of the genetic architecture of male inflorescence is essential to achieve the optimum inflorescence size for maize breeding. In this study, we used approximately eight thousand inbreds, including both linkage populations and association populations, to dissect the genetic architecture of male inflorescence. The linkage populations include 25 families developed in the U.S. and 11 families developed in China. Each family contains approximately 200 recombinant inbred lines (RILs). The association populations include approximately 1000 diverse lines from the U.S. and China. All inbreds were genotyped by either sequencing or microarray. Inflorescence size was measured as the tassel primary branch number (TBN) and tassel length (TL). A total of 125 quantitative trait loci (QTLs) were identified (63 for TBN, 62 for TL) through linkage analyses. In addition, 965 quantitative trait nucleotides (QTNs) were identified through genomewide study (GWAS) at a bootstrap posterior probability (BPP) above a 5% threshold. These QTLs/QTNs include 24 known genes that were cloned using mutants, for example *Ramosa3* (*ra3*), *Thick tassel dwarf1* (*td1*), *tasselseed2* (*ts2*), *liguleless2* (*lg2*), *ramosa1* (*ra1*), *barren stalk1* (*ba1*), *branch silkless1* (*bd1*) and *tasselseed6* (*ts6*). The newly identified genes encode a zinc transporter (e.g. GRMZM5G838098 and GRMZM2G047762), the adapt in terminal region protein (e.g. GRMZM5G885628), O‐methyl‐transferase (e.g. GRMZM2G147491), helix‐loop‐helix (HLH) DNA‐binding proteins (e.g. GRMZM2G414252 and GRMZM2G042895) and an SBP‐box protein (e.g. GRMZM2G058588). These results provide extensive genetic information to dissect the genetic architecture of inflorescence size for the improvement of maize yield.

## Introduction

The male inflorescence (tassel) is an indispensable organ for maize production because it provides pollen for hybridization (Upadyayula *et al*., 2006). Although well‐developed male inflorescences have been observed in teosintes (Piperno and Flannery, 2001), they were reduced during maize domestication and improvement (Duvick and Cassman, 1999) because a larger male inflorescence would compete for photosynthate or contribute to the shading effect (Crue and Wasson, 1996). In fact, varieties with an ideal male inflorescence can produce sufficient pollen and transform more energy into kernels (Eveland *et al*., 2014; Upadyayula *et al*., 2006). Brewbaker (2015) reported a significant decrease in male inflorescence‐related traits, such as tassel primary branch number (TBN) and tassel length (TL), from typical landraces to temperate adapted lines. The male inflorescence is controlled by a large number of genetic loci/genes (Tanaka *et al*., 2013); however, knowledge of its molecular basis remains poor (Eveland *et al*., 2014). Therefore, an in‐depth investigation of the genetic loci/genes that control male inflorescence could provide information for determining the molecular mechanisms of its variation and could improve breeding strategies for enhancing maize grain yield.

There are considerable efforts to map quantitative trait loci (QTLs) for male florescence‐related traits (Berke and Rocheford, 1999; Chen *et al*., 2014; Mickelson *et al*., 2002; Upadyayula *et al*., 2006). However, only limited number of QTLs were identified because only two parental inbreds were selected in each population, which resulted in a limited number of recombination events. To further dissect the genetic basis of the male inflorescence, Yang *et al*. (2014) used the GWAS method in a diverse panel and identified 30 TBN‐ and 33 TL‐related genetic loci, which significantly improved the resolution compared with previous studies. However, the use of GWAS in diverse panels was challenged by the false discoveries caused by the population structure and unequal relatedness among individuals (Zhang *et al*., 2010).

Thus, McMullen *et al*. (2009) employed a different design, that is the nested association mapping population (US NAM) to dissect genetic architecture of complex traits (Buckler *et al*., 2009) using joint‐linkage analysis. Furthermore, effective and reliable results were obtained from GWAS in these populations (Tian *et al*., 2011). Using this US NAM population, many genetic loci associated with different traits of agronomic importance were identified (Brown *et al*., 2011; Buckler *et al*., 2009; Cook *et al*., 2012; Kump *et al*., 2011; Peiffer *et al*., 2014; Tian *et al*., 2011). In particular, Brown *et al*. (2011) detected 39 TBN‐ and 37 TL‐related QTLs and also identified 241 TL‐related QTNs and 325 TBN‐related QTNs. In their study, 13 male inflorescence‐related genes that were cloned using mutant genetics were validated with TBN‐or TL‐related QTNs falling within 1 centi‐Morgen (cM) of these genes. The NAM populations provide high power for the dissection of the genetic architecture of the male inflorescence of maize. However, the mapping resolution is less desirable for gene cloning due to limited number of recombination events.

Until now, only a few genes associated with the male inflorescence have been cloned, for example *Thick tassel dwarf1* (*td1*) (Bommert *et al*., 2005). *Barreninflorescence*2 (*bif2*) regulates axillary meristem development in the maize inflorescence (McSteen and Hake, 2001). *Ramosa1* (*ra1*) (Vollbrecht *et al*., 2005) and *ramosa3* (*ra3*) (Satoh‐Nagasawa *et al*., 2006) are associated with the male inflorescence, as well as the *indeterminate1 mutation* (*id1*) (Aukerman and Amasino, 1998), *flowering1* (*dlf1*) and *leafy1* (*lfy1*) (Colasanti *et al*., 1998), *knotted1*(*kn1*) (Bolduc *et al*., 2012), *branch angle defective 1* (*bad1*) (Bai *et al*., 2012) and two SBP‐box transcription factor genes of *unbranched2* (*ub2*) and *ub3* (Chuck *et al*., 2014).

Recently, with the development of high‐throughput sequencing platforms, Eveland *et al*. (2014) identified 164 genes that were specifically expressed in the male inflorescence using transcriptional expression profile analysis of*ra1*‐R, *ra2*‐R and *ra3*‐*fea1* introgressed families with an identical genetic background of B73. They established a regulatory model and provided additional proof for validating the gene function of the male inflorescence. However, the understanding of the male inflorescence remains limited due to complex molecular networks (Eveland *et al*., 2014) and a limited number of parental lines used previously (Brown *et al*., 2011; Chen *et al*., 2014; Mickelson *et al*., 2002; Upadyayula *et al*., 2006). Therefore, more parental lines with wider genetic diversity are desirable, and a combination of joint‐linkage mapping, GWAS and transcriptional expression profile analysis would be better for dissecting the genetic basis of the male inflorescence in maize.

In this study, we constructed another NAM population (CN NAM) using Huangzaosi (HZS) as the common parental line, with larger male inflorescence size (TBN of 13.93, TL of 29.06) compared with that of B73 (TBN of 10.24, TL of 28.76), which can provide abundant pollen for hybridization in breeding practice. The other 11 parental lines of CN NAM were from different heterotic groups of Reid, Tangsipingtou (TSPT), Lancaster, and the P and Tem‐tropic I groups (Wu *et al*., 2014), which would provide some new allelic variations. In addition, one global association panel (AP) of 945 diverse lines was collected, which could maximize the genetic diversity of the maize germplasm. The objective of this study was to identify the QTLs, QTNs and genes associated with TBN and TL by combining CN NAM, US NAM and AP to provide extensive genetic information for dissecting their potential molecular mechanisms.

## Results

### Phenotypic variations

The phenotypes of TBN and TL are shown in Tables 1, S2 and Figure S2. The results indicated that there were abundant phenotypic variations in the experimental materials, which benefited the dissection of the genetic architecture of the male inflorescence. Broad‐sense heritability ranged from 0.83 to 0.92 for TBN, and 0.69 to 0.85 for TL. A correlation analysis of TBN and TL showed significantly positive associations among different environments, with a *P* value less than 1 × 10^−4^ (Table S3). Therefore, a single best linear unbiased predictor (BLUP) value was calculated for TBN and TL across all locations.

**Table 1:** Summary of genetic loci controlling tassel‐related traits

Trait	Population	Mean	Range	Broad‐sense heritability	No. of QTL	PVE(%)	No. of QTNs	No. of common QTL^a^
Tassel length (TL) (cm)	USNAM parental lines	33.16	22.60–40.85	–				
	CNNAM parental lines	29.55	18.00–43.50	–				
	USNAM	32.70	3.80–58.50	0.69	37	64.35	106	53/106
	CNNAM	29.26	9.80–53.00	0.85	25	67.99	39	27/39
	CN&US NAM	29.43	3.80–58.50	–	–	–	253	139/253
	US AP	29.00	6.20–59.60	0.75	–	–	0	0
	CN AP	29.86	11.90–48.91	0.85	–	–	0	0
	CN&US AP	30.88	6.20–59.60	0.80	–	–	19	9/19
	Common				8			15/253
Tassel primary branch number (TBN)	US‐NAM parental lines	12.09	5.80–24.00	–				
	CN‐NAM parental lines	11.55	2.75–25.00	–				
	US‐NAM	9.42	1.00–41.00	0.90	35	65.11	163	87/163
	CN‐NAM	11.84	1.00–39.80	0.92	28	73.03	32	17/32
	CN&US NAM	10.63	1.00–41.00	–	–	–	328	184/328
	US AP	10.96	1.00–35.00	0.91	–	–	0	0
	CN AP	9.33	1.00–38.80	0.83	–	–	11	2/11
	CN&US AP	10.15	1.00–38.80	0.87	–	–	15	7/15
	Common				13			29/328, 3/15

a Data on the left of oblique line refer to No. of QTNs, which were located in QTL intervals identified by the joint‐linkage strategy. Data on the right of oblique line refer to total number of all QTNs identified through the GWAS strategy.

### Genetic loci controlling phenotypic variations

**Figure 1:**
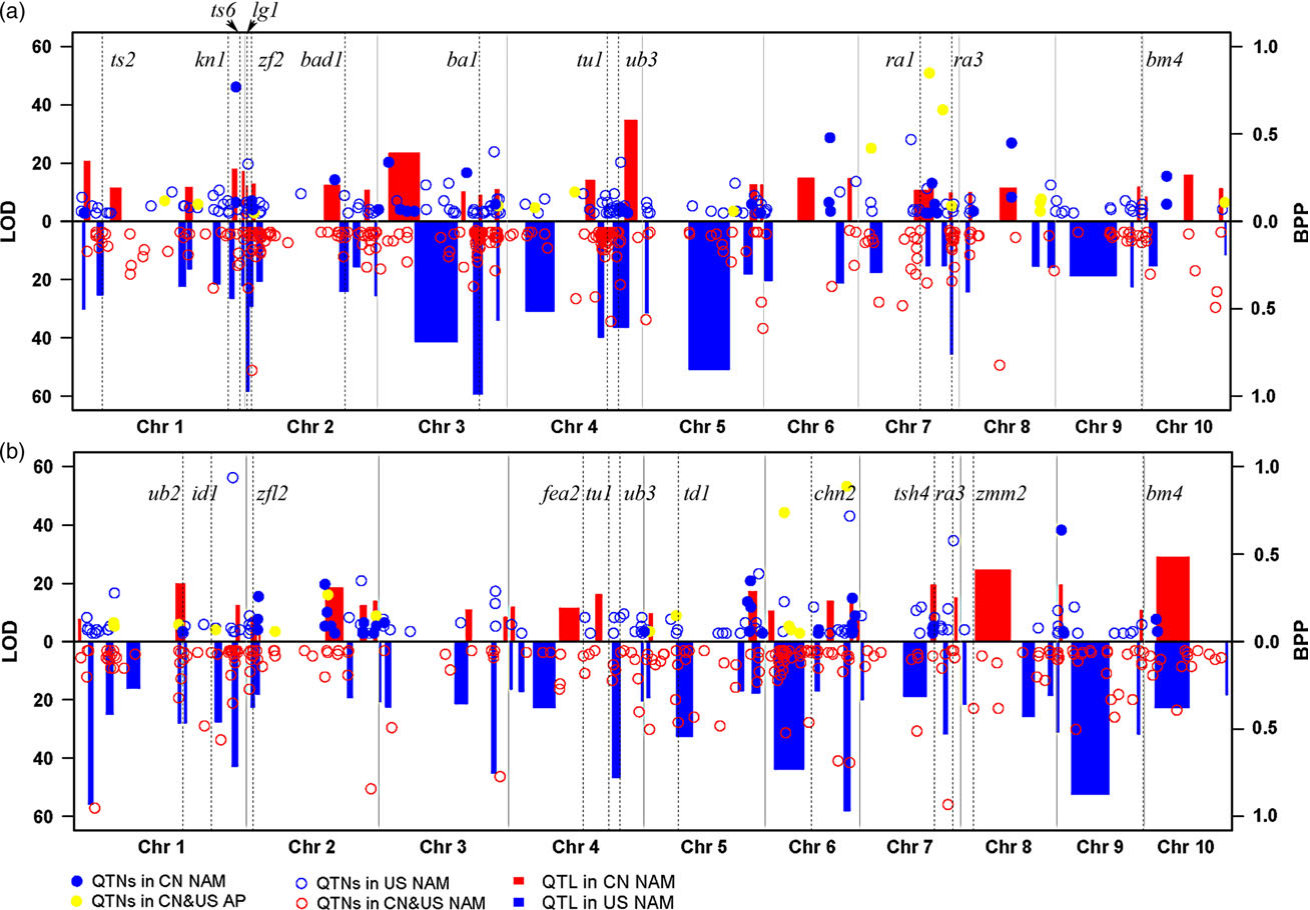
Genetic loci controlling phenotypic variations of tassel‐related traits. ‘a’ showed the genetic loci significantly associated with tassel primary branch number (TBN), and ‘b’ showed the genetic loci significantly associated with tassel length (TL). The rectangles filled with red and blue colour were quantitative trait loci (QTL) identified in CN NAM and US NAM, respectively, with rectangle width to be QTL interval supported by joint‐linkage. Logarithm of odds (LOD) value of QTL was ruled on the left of each plot. Circles and dots with different colours of lines represented QTNs found in different populations, independently. Dashed lines showed tassel‐related genes cloned using maize mutants, previously. ‘QTL in CN NAM’ and ‘QTL in US NAM’ meant QTL identified in the China nested association mapping population and US nested association mapping population, respectively. ‘QTNs in CN&US AP’, ‘QTNs in CN&US NAM’, ‘QTNs in US NAM’, ‘QTNs in CN NAM’ and ‘QTNs in CN AP’ represented quantitative trait nucleotides (QTNs) identified in a combination of Chinese and USA association panels, a combination of Chinese and USA nested association mapping populations, the USA nested association mapping population, the Chinese nested association mapping population and the Chinese association panel, respectively.

For CN NAM, 28 TBN‐related QTLs were detected (Table 1 and Fig. 1a), 12 of which were shared in single families (Fig. S3); the average phenotypic variation explained (PVE) value was 10.19%, and this value varied from 5.69% to 25.33% (Table S5). A total of 25 TL‐related QTLs were detected (Table 1 and Fig. 1b), 13 of which were shared in single families (Fig. S4), with an average PVE value of 11.46% and a range of 5.92% to 30.02% (Table S5).

For US NAM, 35 TBN‐related QTLs were identified (Table 1 and Fig. 1a), 29 of which were shared in single families (Fig. S3); the average PVE value was 10.73%, and this varied from 3.75% to 39.57% (Table S6). A total of 37 TL‐related QTLs were identified (Table 1 and Fig. 1b), 22 of which were shared in single families (Fig. S4); the average PVE value was 10.58%, and this varied from 4.57% to 31.12% (Table S6).

**Figure 2:**
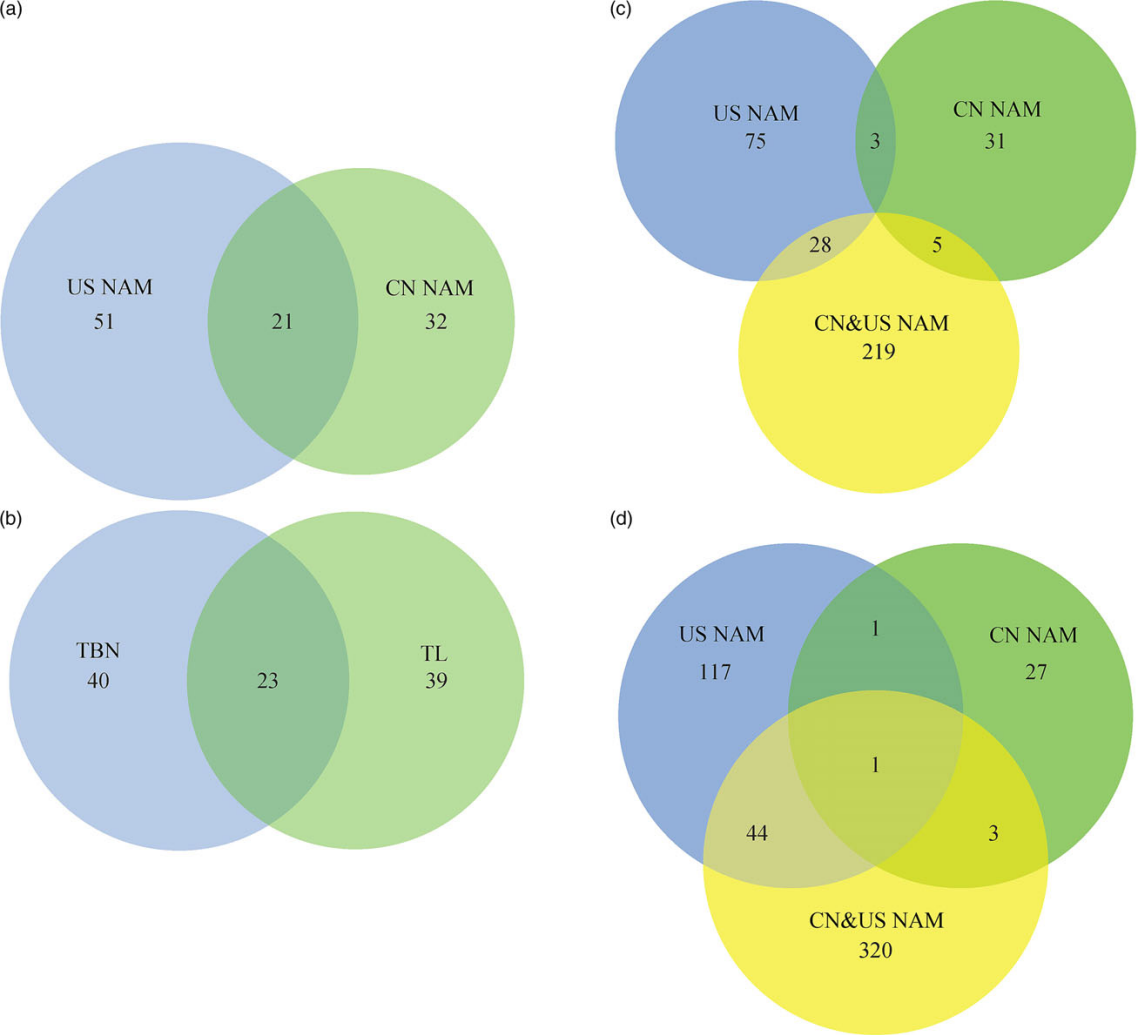
Venn diagram of genetic loci comparison. Plot ‘a’ showed the comparison of QTL supported by joint‐linkage among CN NAM and US NAM. Plot ‘b’ showed the comparison of QTL supported by joint‐linkage among TL and TBN. Plots ‘c’ and ‘d’ represent the comparison of QTNs associated with TL and TBN among CN NAM, US NAM and CN&US NAM respectively.

The comparison analysis of the joint‐linkage analysis results between CN and US NAM (Fig. 2a) showed that 32 tassel‐related QTLs were observed specifically in CN NAM. A total of 51 tassel‐related QTLs were identified specifically in US NAM. A total of 21 QTLs, including 13 TBN‐ and 8 TL‐associated QTLs, were identified as common to both CN and US NAM. In addition, there were 23 common QTLs between TL and TBN (Fig. 2b).

The GWAS results showed that a total of 549 TBN‐related QTNs with BPP values greater than or equal to 0.05 at a *P* value less than 5.46 × 10^−6^ were identified. The use of CN&US NAM showed the largest detection power compared with any other population, where 328 QTNs were detected compared with 163 in US NAM, 32 in CN NAM, 15 in CN&US AP, 0 in US AP, and 11 in CN AP. Similarly, a total of 416 TL‐related QTNs were identified with BPP values greater than or equal to 0.05 at a *P* value less than 5.66 × 10^−6^. CN&US NAM also showed the largest detection power (252 QTNs found) compared with 106 in US NAM, 39 in CN NAM, 19 in CN&US AP, and 0 in US AP and CN AP (Table S4). The comparison analysis of the GWAS results from CN, US and CN&US NAM showed that none of the common QTNs associated with TL were found in the three NAM populations (Fig. 2c). However, 28 TL‐related QTNs were shared between US and CN&US NAM, 5 were shared between CN and CN&US NAM, and 3 were shared between CN and US NAM (Fig. 2c). In addition, there was one common QTN associated with TBN in CN, US and CN$US NAM, 45 QTNs were shared between US and CN&US NAM, 2 were shared between CN and US NAM, and 4 were shared between CN and CN&US NAM (Fig. 2d).

### Overlap between QTNs and QTLs

**Figure 3:**
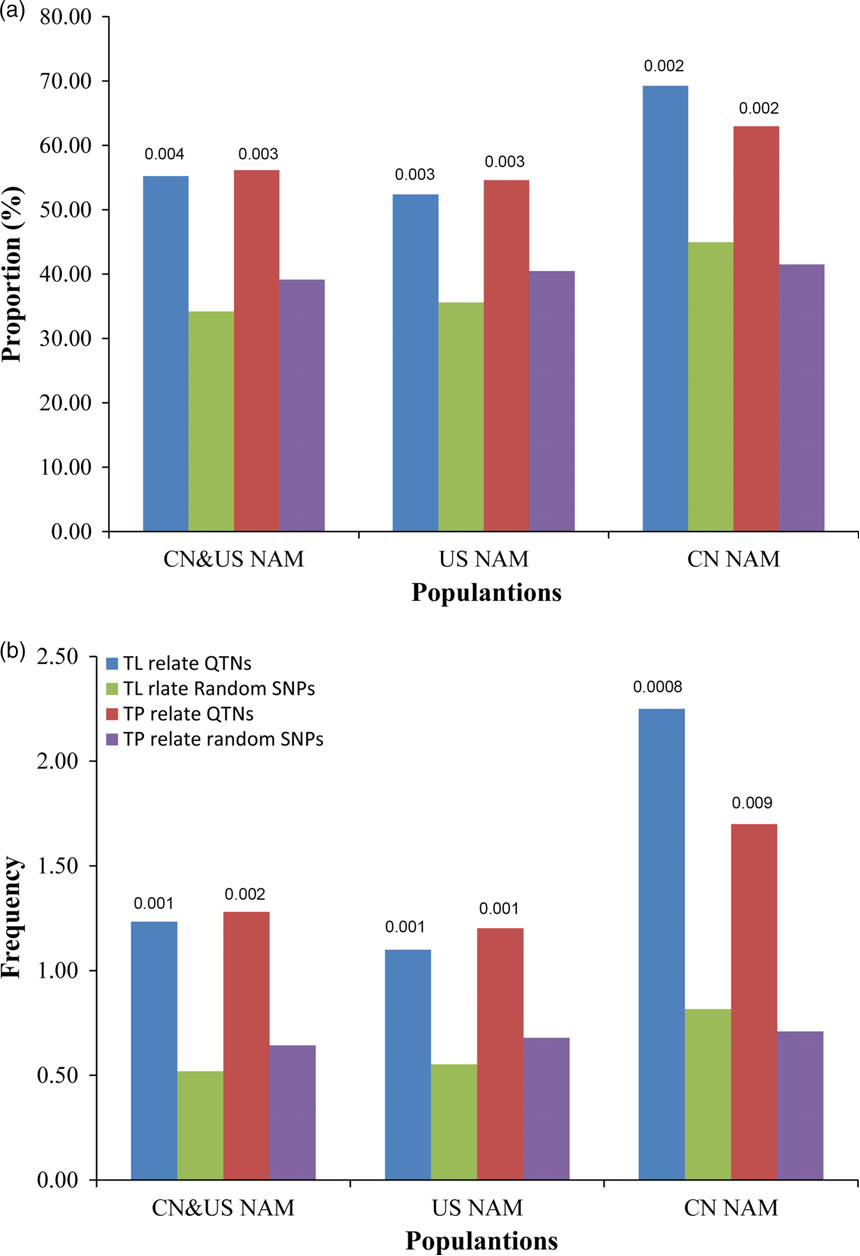
Overlapping between QTNs and QTL identified by joint linkage. Rectangles filled with dark‐blue and dark‐red meant observed values for TL and TBN, respectively. Rectangles filled with olive and purple meant random values for TL and TBN, respectively. Plot ‘a’ showed the proportion of QTNs fell within QTL intervals. And plot ‘b’ showed the ratio of intra‐QTL number versus that of inter‐QTL intervals.

Significant overlaps existed between the QTNs detected using GWAS and the QTLs detected using the joint‐linkage analysis (Fig. 3), with *P* value less than 0.001 between actual proportion and expected ones. Detailed information on the QTNs and QTLs are listed in Table S4. For CN&US NAM, the frequency of the observed SNPs falling within the QTLs was significantly higher than that of random SNPs, and similar results were found in both CN NAM and US NAM (Fig. 3b). In addition, for the TBN‐related QTNs, 300 of 549 QTNs were co‐located within the QTL intervals, a finding that was supported by the joint‐linkage analysis, with 78 QTNs specific only to the CN NAM QTL intervals, 179 QTNs specific only to the US NAM QTL intervals, and 43 QTNs shared between the CN NAM and the US NAM QTL intervals. Among the 416 TL‐related QTNs, 243 were co‐located within the QTL intervals, with 61 QTNs specific only to the CN NAM QTL intervals, 161 QTNs specific only to the US NAM QTL intervals and 21 QTNs shared between the CN NAM and US NAM QTL intervals. These loci would be more robust and closely linked to the causal effects of variations in the male inflorescence.

### QTN enrichment in candidate genes

**Figure 4:**
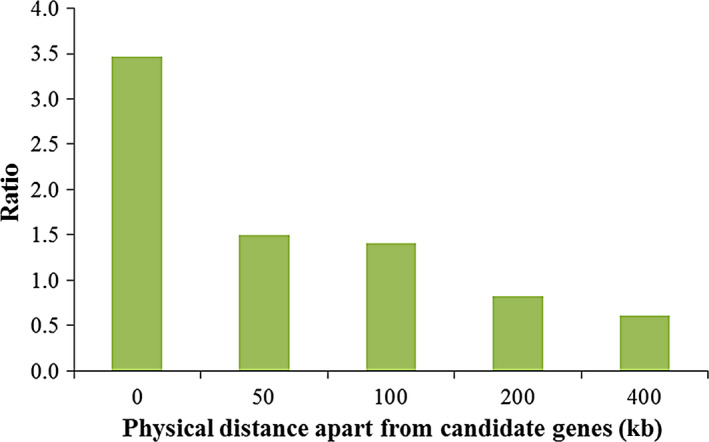
QTNs enrichment in candidate genes. Significantly overlapping was found.

The QTNs identified in association studies only indicated the linkage disequilibrium between the SNPs and the phenotypes. To build connections between these associated SNPs and the potential causal genes, we conducted the enrichment studies on candidate genes. The results showed significant QTN enrichment within the candidate genes, with 3.47 *vs* 1 between the actual QTNs and the random SNPs falling within 0 kb of the candidate genes, 1.5 *vs* 1 between the actual QTNs and the random SNPs falling within 50 kb of the candidate genes, 1.4 *vs* 1 between the actual QTNs and the random SNPs falling within 100 kb of the candidate genes, 0.82 *vs* 1 between the actual QTNs and the random SNPs falling within 200 kb of the candidate genes, and 0.61 *vs* 1 between the actual QTNs and the random SNPs falling within 400 kb of the candidate genes (Fig. 4). In addition, 113 candidate genes (Fig. 1, Table 2 and Table S4) with peak signal SNPs falling within 100 kb of them are listed, among which 24 male inflorescence‐related genes were previously cloned using mutant genetics. For example, the SNP of S7_166858805 with a *P* value of4 × 10^−10^ falls within 100 kb of the *ra3* gene (Fig. 1a and b), and the SNP of S1_46687160 with a *P* value of 5.3 × 10^−10^ falls within 6 kb of the *tasselseed2* (*ts2*) gene (Fig. 1a and Table S4). Additionally, we identified 23 new candidate genes that were remarkably associated with tassel phenotypic variations and that are specifically expressed in the male florescence (Eveland *et al*., 2014). These candidate genes are involved in tassel development and provide positive information for dissecting the genetic basis of tassel architecture variations.

**Table 2:** Candidate genes involved in tassel development

SNP	Trait	Chr.	Position	Falling in CN NAM QTL interval	Falling in US NAM QTL interval	Name of gene/id	Expression specific in tassel
S1_16777433	TL	1	16777433	Yes		GRMZM5G838098	Yes
S1_40420190	TP	1	40420190		Yes	*ibp2*	
S1_46687160	TP	1	46687160		Yes	*ts2*	
S1_52095937	TL	1	52095937		Yes	*cal3*	
S1_61020552	TL	1	61020552		Yes	GRMZM5G885628	Yes
S1_64014376	TL	1	64014376	Yes		*pdlk1*	
S1_119091806	TP	1	119091806			*aic1*	
S1_180307882	TL	1	180307882	Yes	Yes	*bx9*	
S1_187646982	TL	1	187646982	Yes	Yes	GRMZM2G141320	Yes
S1_188063921	TL	1	188063921	Yes	Yes	*ub2*	
S1_199964537	TP	1	199964537	Yes	Yes	*hm1*	
S1_238819120	TL	1	238819120			*id1*	
S1_258800198	TP	1	258800198			*tbp1*	
S1_267366245	TP	1	267366245			*gln2*	
S1_269358662	TL	1	269358662			*phyA1*	
S1_271913038	TP	1	271913038			*kn1*	
S1_276255020	TL	1	276255020			*exg1*	
S1_277844691	TL	1	277844691		Yes	*mta1*	
SYN13726	TL	1	279007172		Yes	*cka2*	
S1_281100720	TL	1	281100720		Yes	*lem1*	
S1_286514978	TP	1	286514978			*vp8*	
S1_288396001	TP	1	288396001			GRMZM2G046163	Yes
S1_292326337	TP	1	292326337			*ts6*	
S1_299609447	TP	1	299609447			*akin1*	
S2_4101480	TP	2	4101480	Yes	Yes	*lg1*	
S2_7099518	TP	2	7099518			*sgb101*	
S2_10568439	TL	2	10568439	Yes		GRMZM2G049538	Yes
S2_12687587	TP	2	12687587		Yes	*zfl2*	
S2_14467324	TP	2	14467324	Yes		*CDPK1*	
S2_16037854	TP	2	16037854	Yes		*hon101*	
S2_17230208	TP	2	17230208	Yes		*nfd102*	
S2_36359741	TP	2	36359741			*mas1*	
S2_41479516	TP	2	41479516			*grf1*	
S2_44203460	TP	2	44203460			GRMZM2G470882	Yes
S2_44697923	TP	2	44697923			*ts1*	
S2_47088159	TP	2	47088159			*opr5*	
S2_51005257	TL	2	51005257			*gpm300*	
S2_54458735	TP	2	54458735			*hrg1*	
S2_118615859	TL	2	118615859			GRMZM2G147491	Yes
S2_172491959	TP	2	172491959		Yes	*akh2*	
S2_176647351	TP	2	176647351		Yes	*vdac1a*	
S2_179817326	TP	2	179817326		Yes	*bad1*	
S2_184964564	TP	2	184964564		Yes	GRMZM2G414252	Yes
S2_213205215	TP	2	213205215			GRMZM2G075244	Yes
S2_224064908	TP	2	224064908			*rrb1*	
S2_229451557	TP	2	229451557			*pls1*	
S2_235802795	TP	2	235802795			*zap1*	
S3_53737606	TP	3	53737606	Yes		*lg3*	
S3_137240173	TP	3	137240173		Yes	*zag2*	
S3_176567842	TP	3	176567842		Yes	*lg2*	
S3_179732428	TP	3	179732428		Yes	*taf1*	
S3_182623814	TP	3	182623814		Yes	*gpm298*	
S3_183142036	TP	3	183142036	Yes	Yes	*ba1*	
S3_184268939	TP	3	184268939	Yes	Yes	*expa1*	
S3_206845615	TL	3	206845615		Yes	GRMZM2G447632	Yes
S3_212025832	TP	3	212025832	Yes		*bzip1*	
PZE‐103167491	TP	3	216305962	Yes	Yes	*a1*	
S4_133453055	TL	4	133453055			*fea2*	
S4_143822114	TL	4	143822114			*pip2c*	
S4_153535768	TP	4	153535768	Yes		*pip1c*	
S4_166926544	TP	4	166926544		Yes	GRMZM2G362823	Yes
S4_167185789	TP	4	167185789		Yes	*gln5*	
S4_170970713	TP	4	170970713		Yes	*prh1*	
S4_175723750	TP	4	175723750			*lkrsdh1*	
S4_177599861	TP	4	177599861			*acco20*	
S4_178821367	TP	4	178821367			*tu1*	
S4_180444282	TP	4	180444282			*rpl29*	
S4_181871621	TP	4	181871621			*gol1*	
S4_188156035	TP	4	188156035			*rcph2*	
S4_188415555	TP	4	188415555			*zcn6*	
S4_199365817	TP	4	199365817		Yes	*ub3*	
S4_200032051	TL	4	200032051			GRMZM2G465165	Yes
S4_233628414	TP	4	233628414			GRMZM2G015419	Yes
S5_30165927	TL	5	30165927			*eif7*	
S5_60804319	TL	5	60804319		Yes	*cdpk1*	
S5_61671700	TL	5	61671700		Yes	*td1*	
S5_83868038	TP	5	83868038			*hppd1*	
S6_24606844	TL	6	24606844		Yes	*gsh1*	
S6_80949310	TL	6	80949310			*prc1*	
S6_82616120	TL	6	82616120			*chn2*	
S6_121499756	TP	6	121499756			*zcn11*	
S6_135884240	TP	6	135884240		Yes	*sod3*	
S6_153206873	TP	6	153206873	Yes		GRMZM2G047762	Yes
PZE‐107005664	TP	7	3892964			GRMZM2G450866	Yes
S7_22013295	TP	7	22013295		Yes	*sid1*	
S7_24412425	TP	7	24412425		Yes	*crt2*	
S7_110782210	TP	7	110782210	Yes		*ra1*	
S7_133158462	TL	7	133158462	Yes		*tsh4*	
S7_162978078	TP	7	162978078			*tif5A*	
S7_166371210	TP	7	166371210	Yes	Yes	*rps6*	
S7_166858805	TP	7	166858805	Yes	Yes	*ra3*	
S7_168576142	TP	7	168576142			*PDK2*	
S7_171933622	TP	7	171933622			*bd1*	
S8_6359026	TL	8	6359026		Yes	GRMZM2G058404	Yes
S8_22641462	TL	8	22641462			*zmm2*	
S8_100398177	TP	8	100398177	Yes		*act1*	
PZE‐108089142	TP	8	146114537			GRMZM2G070837	Yes
PZA02403.12	TP	8	153895493			GRMZM5G833406	Yes
S8_157747137	TL	8	157747137		Yes	GRMZM2G307756	Yes
S8_158571117	TL	8	158571117		Yes	*elm1*	
S8_173079005	TL	8	173079005		Yes	*gst1*	
S9_61296310	TL	9	61296310		Yes	*pep1*	
S9_134997977	TP	9	134997977			*phyB2*	
S9_135481090	TL	9	135481090			*NAS1*	
S9_139749854	TL	9	139749854			*IBP1*	
PZE‐109103089	TL	9	146685045		Yes	*ZMM8*	
S9_151732656	TL	9	151732656	Yes		*gst9*	
S9_153435468	TP	9	153435468			GRMZM2G075563	Yes
S9_154650092	TP	9	154650092			*bm4*	
S9_155578330	TP	9	155578330			GRMZM2G161827	Yes
S10_76506157	TL	10	76506157	Yes	Yes	GRMZM2G042895	Yes
S10_84229674	TL	10	84229674			*orp2*	
PZE‐110103696	TP	10	146292761			GRMZM2G058588	Yes

### Comparison of GWAS among different populations

**Figure 5:**
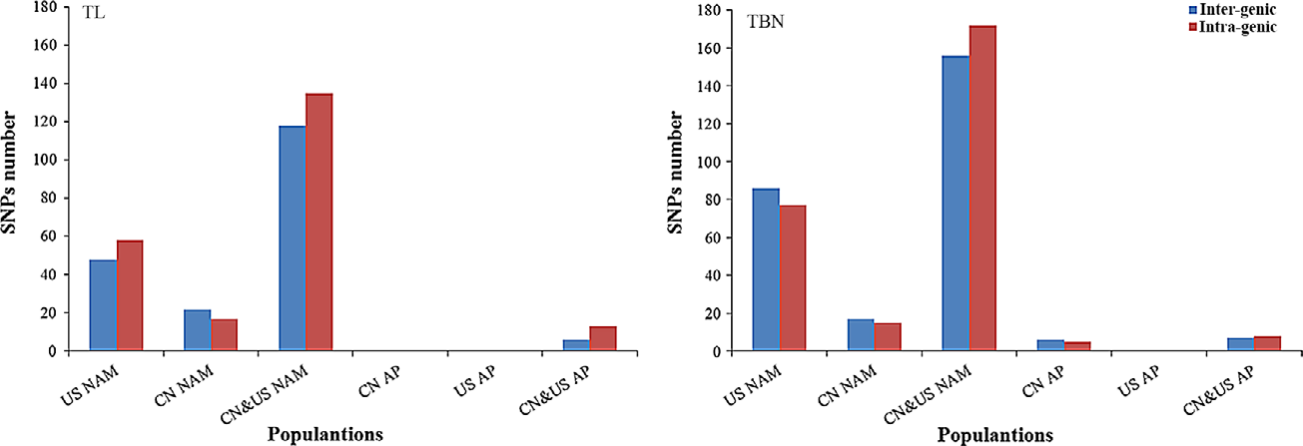
Comparison of detection resolution. ‘TL’ was the abbreviation of tassel length, and ‘TBN’ was the abbreviation of tassel primary branch number.

The GWAS results using different populations were compared (Table 1, Fig. 5 and Table S4). For the TL trait, 252 QTNs were identified when using the largest mapping population of CN&US NAM (6595 RILs), which showed the highest number of QTNs among the three NAM populations; only 106 QTNs were identified in US NAM using 4623 RILs, and 39 QTNs were identified in CN NAM using 1972 RILs. In addition, the American panel with 280 lines and the Chinese panel with 665 lines showed no detection of TL‐related QTNs when using the two populations independently; however, 19 TL‐related QTNs were identified when the two APs were combined (Fig. 5 TL). For the TBN trait, 328 QTNs were identified using CN&US NAM, which also showed the highest number of QTNs compared with the other two NAM populations; no QTN was found in US AP, whereas 11 QTNs were found in CN AP and 15 QTNs were found in CN&US AP (Fig. 5 TBN).

## Discussion

### Abundant phenotypic variations in the male inflorescence

The US NAM involved included 26 diverse parental inbreds, which maximized the maize phenotypic diversity in the population (McMullen *et al*., 2009). In the study conducted by Brown *et al*. (2011), the population had a large phenotypic variation in TL, ranging from 3.80 to 58.50 cm, and in TBN, ranging from 1.00 to 41.00 (Table 1), which was considerably higher than in the bi‐parental populations of ILP× B73 (Upadyayula *et al*., 2006) and Chang 7–2 × 787 (Chen *et al*., 2014). Because greater phenotypic variation would be beneficial for dissecting the genetic architecture of the male inflorescence, more experimental materials, including 1732 RILs of CN NAM and 945 diverse lines of CN&US AP, were added in the present study. These lines were evaluated across 13 environments, and the results showed that the phenotypic variations in TL and TBN were remarkably higher than those in any single population described previously (Brown *et al*., 2011; Chen *et al*., 2014; Upadyayula *et al*., 2006).

### Common and adaptive loci affect male inflorescence conjointly

The results obtained in this study revealed some common and adaptive genetic loci in both CN NAM and US NAM. A total of 25 common QTLs (17 for TBN and 8 for TL) between CN NAM and US NAM, 32 specific QTLs in CN NAM (15 for TBN and 17 for TL) and 51 specific QTLs in US NAM (22 for TBN and 29 for TL) were identified (Fig. 2a,b). In addition, 64 QTNs were co‐located within 25 common QTL intervals, 340 QTNs fell within 51 US NAM QTL intervals, and 139 QTNs fell within 32 CN NAM QTL intervals (Table S4). A common genetic mechanism for the male inflorescence is perhaps under standable because some parental inbreds of CN NAM were clustered into the same group as that of US NAM (Fig. S1). The adaptive genetic loci that were detected might be explained by the different germplasm background of CN NAM and US NAM. The common parent of US NAM, that is B73, which is a representative inbred of the SS group, is widely used in the U.S. maize breeding programme (van Heerwaarden *et al*., 2012), with TBN of 10.24 ± 4.94 (Table S2). The other 25 parents of US NAM, including 13 inbreds from tropical regions (TBN of 11.57 ± 5.32), 9 inbreds from temperate regions (TBN of 6.55 ± 5.96) and 3 from Europe (TBN of 6.25 ± 5.78), showed larger variations in the male inflorescence, that is the tropical lines showed the largest TBN value, which is consistent with the report of higher diversity in tropical maize germplasm (Yan *et al*., 2009). However, according to Mir *et al*. (2013), maize in Eastern Asia is closely related to the Mexican highland landraces. After its initial introduction into China, probably 500 years ago, maize has extended to wide regions of this country and has formed a number of landraces with strong adaptability.

Huangzaosi (HZS) is a typical inbred line that was derived from local Chinese landraces. This line has well‐developed tassels to provide abundant pollen under different ecological environments and has an average TBN of 13.93 ± 4.34, which is significantly higher than that of B73 (Table S2). The feature of large tassels is one reason why HZS is more adaptive than B73 in the Chinese maize breeding programme, especially in the Huanghuihai region. HZS has become a foundation line in the TSPT group, which is a popular heterotic group used in China (Wang *et al*., 2011). The other parents of CN NAM included 5 inbreds from TSPT, 3 from modified Reid, 1 from Lancaster and 2 from Tem‐tropic I, some of which were derived from U.S. germplasm (Wang *et al*., 2008; Wu *et al*., 2014).Thus, although the number of QTL detected in CN NAM was less than that in US NAM due to a narrower genetic diversity of the CN NAM population, a joint analysis of the two NAM populations could reveal the common and adaptive genetic loci controlling the male inflorescence of maize. This information may provide interesting clues for maize improvement programmes with different breeding targets aimed at different environments.

### Larger and more diverse mapping populations improved the detection resolution

Differences in the population size and genetic diversity would be the main factors for different detection resolutions and power because larger population sizes and allelic diversity would contain a greater number of recombinants (McMullen *et al*., 209). In this study, the two independent NAM populations were used in the joint‐linkage analysis, resulting in 72 and 53 male inflorescence‐related QTLs that were identified in US NAM using 4623 RILs and CN NAM using 1972 RILs, respectively (Table S5 and S6). In addition, 269, 71 and 580 male inflorescence‐related QTNs were identified in US NAM, CN NAM and CN&US NAM, respectively (Table S4). For the association panels, CN&US AP with 945 diverse lines showed the highest resolution with 34 tassel‐associated QTNs, whereas CN AP with 665 inbreds showed the second highest resolution with 11 tassel‐associated QTNs, and US AP with 280 lines had no detection power (Table S4). The results suggest that larger and more diverse populations may benefit the dissection of the genetic architecture of complex traits, irrespective of the linkage analysis and GWAS.

### Extensive genetic loci and candidate genes involved in the male inflorescence

To explore the biological connection of the QTLs/QTNs to the causal genes, we examined the genes that our QTLs/QTN landed exactly and the nearby candidate genes. The annotation of biological information (MaizeGDB: http://www.maizegdb.org) showed that a total of 503 QTNs fell within genes, including those encoding 57 transcription factors (TF), 29 kinases, and 22 ubiquitin–proteasome system (UPS) and 395 unknown types (Table S4). Among the 13 TBN‐ and TL‐related genes validated by Brown *et al*. (2011) with peak signal SNPs falling within 1 cM of the genes, 8 genes were identified in this study, with peak signal SNPs falling within 200 kb of the genes (Table 2), including *kn1* (Bolduc *et al*., 2012), *lg2* (Walsh *et al*., 1998), *zfl2* (Bomblies *et al*., 2003), *td1* (Bommert *et al*., 2005), *ts2* (Kindiger *et al*., 1995), *ra1* (Vollbrecht *et al*., 2005), *ra3* (Satoh‐Nagasawa *et al*., 2006) and *fasciated ear2* (*fea2*) (Taguchi‐Shiobara *et al*., 2001). In addition, another 16 known TBN‐ and TL‐related genes cloned using mutant genetics were also validated in this study, including *ba1* (Barazesh and McSteen, 2008), *bd1* (Chuck *et al*., 2002), *ts6* (Chuck *et al*., 2007), *ts1* (Hultquist and Dorweiler, 2008), *tasselsheath4* (*tsh4*) (Chuck *et al*., 2010), *bad1* (Bai *et al*., 2012), *ub2* and *ub3* (Chuck *et al*., 2014), and *tga1*(Preston *et al*., 2012) (Fig. 1 and Table 2).

More importantly, 23 new candidate genes were identified with peak signal SNPs falling within 200 kb of the genes (Table 2). These genes are specifically expressed in the male inflorescence (Eveland *et al*., 2014). For example, a TIFY domain protein (GRMZM5G838098) that was previously annotated as a zinc transporter and which is highly expressed in the florescence meristem of rice (Nishii *et al*., 2000) was suggested to coordinate tissue growth, modulate lamina size and regulate cell cycle arrest in *Arabidopsis* (White, 2006). The adapt in terminal region protein (GRMZM5G885628) played multiple roles in regulating the soluble NSF attachment protein receptor (SNARE) activity and targeting *via* interaction with other trafficking proteins (Vedovato *et al*., 2009), which indicates a central role in the mechanics of cell growth and development (Blatt *et al*., 2008). Mono‐galactosyl‐diacyl‐glycerol (MGDG) synthase (GRMZM2G141320) was shown to be essential for the synthesis of galactolipids and the development of photosynthetic membranes in *Arabidopsis* (Dubots *et al*., 2010). Tryptophan synthase (GRMZM2G046163) is able to catalyse reaction‐producing indole (Kriechbaumer *et al*., 2008), which is required for tryptophan synthesis for improving the nutritional quality of cereal (Wenefrida *et al*., 2013).

Terpene synthase (GRMZM2G049538) catalyses the biosynthesis of terpene, which defends the plant from insect attack (Schnee *et al*., 2002). O‐methyl‐transferase (GRMZM2G147491) is a key enzyme in the biosynthesis of lignin (Fornale *et al*., 2006), which could increase the strength and stiffness of fibres, improve the efficiency of water transport through the vascular system and protect plants from pathogen attack (Boerjan *et al*., 2003). The helix‐loop‐helix (HLH) DNA‐binding proteins (GRMZM2G414252 and GRMZM2G042895) regulate the biosynthesis of anthocyanin in maize (Tominaga‐Wada *et al*., 2012), which attracts pollinators and seed dispersers and defends plants against abiotic and biotic stresses (Petroni and Tonelli, 2011). Cytochrome (P450) (GRMZM2G075244) affects meristem function (Miyoshi *et al*., 2004). Glutathione S‐transferase (GRMZM2G447632) plays a role in the cellular response to auxins during the normal metabolism of plant secondary products, such as anthocyanins and cinnamic acid (Banerjee and Goswami, 2013). Legume lectin (GRMZM2G465165) contains a conserved residue of carbohydrate recognition domain (CRD), which is associated with organisms from all kingdoms of life (De Hoff *et al*., 2009) and which can coordinate metals (Ca^2+^ and Mn^2+^) (Arason, 1996) and bind to phytohormones such as adenine‐related cytokinins, abscisic acid and gibberellic acid (Bogoeva *et al*., 2004). The SBP‐box protein (GRMZM2G058588) regulates primordia initiation, and family members of the transcription factor genes *ub2* and *ub3* affect TBN (Chuck *et al*., 2014).

These candidate genes may play important roles in male inflorescence development, but their biological functions require further investigation. Although many of QTLs/QTNs identified in this study were enriched for previous candidate gene studies, our study was primary served the first step towards full discoveries of genes controlling male inflorescence.

## Materials and methods

### Plant materials and phenotypic evaluations

A total of 6595 recombinant inbred lines (RILs) were used in this study, including 4623 RILs from US NAM and 1972 RILs from CN NAM. The construction of US NAM and CN NAM has been described previously (McMullen *et al*., 2009). In addition, one global association panel consisting of 945 diverse lines was used in this study, which was collected from the U.S. and China, including one U.S. association panel (US AP) with 280 diverse lines and one Chinese association panel (CN AP) with 665 diverse lines. Detailed information on the NAM parents, CN NAM RILs and AP lines are listed in Table S1 and shown in Figure S1.

For US NAM and US AP, the TBN and TL data were collected across eight environments that were described previously (Brown *et al*., 2011). For CN NAM, TBN and TL were measured in six environments, including Beijing in 2009 and 2010, Xinxiang in Henan Province in 2009 and 2010, and Urumqi in Xinjiang Province in 2009 and 2010. For CN AP, TBN and TL were measured in six environments, including Beijing in 2011, Changchun in Jinlin Province in 2011, Nanchong in Sichuan Province in 2011, Tai'an in Shandong Province in 2011 and Xinxiang in Henan Province in 2011 and 2012. At each location, the parental lines and the RILs were planted according to a randomized experimental design, with a single row plot, a row length of 3 m, 0.6 m between adjacent rows, two replications and 11 plants in each row. TL and TBN were measured 15 days after pollen shedding, according to a method described previously (Brown *et al*., 2011). TL was defined as the length from the base of the first branch to the tip of the tassel, measured in centimetres (cm), and TBN was counted in the branch zone.

### Phenotypic data analysis

Phenotypic data analysis was performed using SAS software (Release 9.3; SAS Institute, Cary, NC), and ANOVA was performed using the PROC GLM model. Pearson correlation analysis was calculated using the PROC CORR model. The broad‐sense heritability (h^2^) for TBN and TL was calculated as follows: $h^{2}=\sigma_{g}^{2}/\left(\sigma_{g}^{2}+\sigma_{ge}^{2}/n+\sigma_{\varepsilon }^{2}/nk\right)$ (Hallauer and Miranda, 1988), where $\sigma_{g}^{2}$ is the genotypic variance, $\sigma_{ge}^{2}$ is the interactive variance of genotype and environment, $\sigma_{\varepsilon }^{2}$ is the error variance, and *n* and k represent the environment and replication number, respectively. The best linear unbiased predictor (BLUP) calculation was performed using the PROC MIXED model, with genotype, location, genotype*location and replications as random effects (Brown *et al*., 2011).

### Joint‐linkage analysis

The RILs from CN NAM and US NAM were genotyped using the method of genotyping by sequencing (GBS) (EIshire *et al*., 2011). High‐density markers (~1 000 000 SNPs) were obtained, and approximately 950 000 high‐quality SNPs were selected to construct a bin map. A total of 5296 and 4932 bin markers were observed for US NAM and CN NAM, respectively (Li *et al*., 2015). To perform the joint‐linkage analysis of TBN and TL, these bin markers were selected independently in US and CN NAM using the stepwise selection procedure in the PROC GLMSELECT model (Brown *et al*., 2011). TBN‐ and TL‐related QTLs were independently mapped in US NAM using 4623 RILs and in CN NAM using 1972 RILs. For US NAM, the family term was introduced into the model, and each of the 5296 marker‐by‐family terms was made available for inclusion. Significance levels for the entry and exit of the model terms were determined by permutation as follows: Phenotypic data were separately permuted against the genotypic data within each family. All 5296 marker‐by‐family terms were tested, and the lowest *P* value was recorded for each permutation. A total of 1000 permutations were performed, and alpha was set at 0.001. For CN NAM, 4932 bin markers were used to perform the joint‐linkage analysis using the same method described for US NAM. For each single bi‐parental family, QTL analysis was performed using the IciMapping software with a modified algorithm of composite interval mapping (ICIM) (Li *et al*., 2007).

### Genomewide association study of tassel‐related traits

The genomewide association study (GWAS) used the association panel (AP) that was genotyped previously using the MaizeSNP50 BeadChip (Cook *et al*., 2012; Wu *et al*., 2014) and the two NAMs that were genotyped as described above. All markers were screened according to the following criteria: (i) the minor allele frequency (MAF) was larger than 0.05, and (ii) the marker had an unambiguous position on the physical map. Subsequently, GWAS analysis was performed independently for US NAM, CN NAM, CN&US NAM, US AP, CN AP and CN&US AP. Approximately 0.5 million SNPs for the NAMs and 44 000 SNPs for AP were selected to conduct the GWAS by using mixed linear model implemented in the GAPIT R package (Lipka *et al*., 2012).

On top of GWAS analysis, we used the subsampling‐based multiple SNPs model (Brown *et al*., 2011; Tian *et al*., 2011). Briefly, 80% of the original entries were sampled in the new subpopulation without replacement, and forward regression was used to fit the SNPs using permutation‐derived significance thresholds. This process was repeated 100 times to obtain a bootstrap posterior probability (BPP) value for each SNP, ranging from 0 to 1, which represented the proportion of samples in which that SNP was selected. Only SNPs with BPP values greater than or equal to 0.05 were considered to be significantly associated with phenotypic variations and were then designated to quantitative trait nucleotides (QTNs).

### Overlapping between QTNs identified using GWAS and QTLs identified by joint‐linkage analyses

To evaluate the overlap between the GWAS and joint‐linkage results, 549 observed TBN‐, 416 observed TL‐associated QTNs and 125 QTLs identified in CN NAM and U.S. NAM were selected to compare whether QTNs filled within QTLs according to the identical physical position. If one QTN filled within QTL, we deduced this QTN was overlapped with relevant QTL, in contrast, none overlapped was existed between QTN and QTL. Then, calculate the actual proportion of overlapped QTNs, with the number of QTNs filled within QTLs divided by the total number of QTNs, which was defined as actual intra‐QTL ratio. In addition, a subset of 965 SNPs was randomly sampled from 0.5 million SNPs across the genome, with 1000 replications. Then, the proportion of random SNPs falling within the QTL intervals was calculated as described above, this was defined as the expected intra‐QTL ratio by chance. Then, a comparison analysis between the actual intra‐QTL ratio and random intra‐QTL ratio was performed using the binomial distribution test, and overlaps between the QTNs and QTLs were recorded as significant when the actual intra‐QTL ratio was significantly higher than the expected intra‐QTL ratio (Tian *et al*., 2011). Beyond that, we have compared the significant differences of (intra‐QTL ratio)/(1 ‐ intra‐QTL ratio) between actual values and expected ones using the binomial distribution test, providing another proof for validating the overlapping between GWAS results and linkage results.

### QTN enrichment in candidate genes associated with tassel traits

To evaluate the QTN enrichment, 203 tassel‐related candidate genes were collected according to previous literature (Bai *et al*., 2012; Brown *et al*., 2011; Chuck *et al*., 2007, 2014; Eveland *et al*., 2014; McSteen and Hake, 2001). These candidate genes included 43 male inflorescence‐related genes cloned using mutant genetics and 164 candidate genes that are specifically expressed in the male inflorescence (Eveland *et al*., 2014). First, we have defined candidate genetic regions as 0, 50, 100, 200 and 400 kb apart from target candidate genes, then evaluate the overlapping between QTNs and relevant candidate genetic regions (CGLs). QTNs falling within CGL according to the identical physical position were overlapped with relevant CGLs that were defined as the actual intra‐CGL, and relevant number was counted. Then, calculate the actual proportion of overlapped QTNs, with the number of QTNs filled within CGL divided by the total number of QTNs, which was defined as actual intra‐CGL ratio. In addition, a subset of 965 SNPs was randomly sampled from 0.5 million SNPs across the genome, with 1000 replications (Brown *et al*., 2011). The proportion of random SNPs falling within CGL intervals was calculated as described above, which was defined as the expected intra‐CGL ratio by chance. Then, a comparison analysis between the actual intra‐CGL ratio and random intra‐CGL ratio was performed using the binomial distribution test, overlaps between the QTNs and CGLs were recorded as significant when the actual intra‐CGL ratio was significantly higher than the expected intra‐CGL ratio (Tian *et al*., 2011), with ratio of actual values divided by expected ones to be more than 1.

### Elimination of potential biases due to population structure and nongenetic effects

As our plant material was developed and measured in both the US and China, it is critical to eliminate the biases due to germplasm and nongenetic effects such as the environmental difference between the US and China. We have modelled our analyses in three layers to eliminate the potential biases. First, the joint‐linkage analyses and the association studies were performed on inbred BLUPs, which were adjusted by locations, and the interaction between inbreds and locations. The phenotypic differences due to nongenetic effects, such as the environmental differences between the US and China, were removed from BLUPs. Second, the joint‐linkage analysis included families as covariates. The QTL effects were based on segregation within family. Third, we derived principal components (PCs) from all genetic markers that cover whole maize genome. We incorporated PCs as covariate in the US‐CN joint GWAS to eliminate the bias due to population structure.

## Supporting information


**Figure S1** Scatter plot of the first three principal components.

**Figure S2** Phenotypic variations of tassel‐related traits.

**Figure S3** QTL associated with tassel primary branch number (TBN) across the 36 RILs families.

**Figure S4** QTL associated with tassel length (TL) across 36 RILs families.

**Table S1** Summary information of the CN NAM and the lines in the association panel.
**Table S2** Tassel traits observed across different environments.
**Table S3** Correlation analysis of tassel traits among different environments.
**Table S4** QTNs associated with tassel traits.
**Table S5** QTL detected in CN NAM by joint‐linkage analysis and validated in single bi‐parental RIL family.
**Table S6** QTL detected in US NAM by joint‐linkage analysis and validated in single bi‐parental RIL family.

## References

[pbi12519-bib-0001] Arason, G.J. (1996) Lectins as defence molecules in vertebrates and invertebrates. Fish Shellfish Immun. 6, 277–289.

[pbi12519-bib-0002] Aukerman, M.J. and Amasino, R.M. (1998) Floral induction and florigen. Cell, 93, 491–494. 9604924 10.1016/s0092-8674(00)81178-2

[pbi12519-bib-0003] Bai, F. , Reinheimer, R. , Durantini, D. , Kellogg, E.A. and Schmidt, R.J. (2012) TCP transcription factor, branch angle deffective1 (bad1), is required for normal tassel branch angle formation in maize. Proc. Natl Acad. Sci. USA, 109, 12225–12230. 22773815 10.1073/pnas.1202439109 PMC3409762

[pbi12519-bib-0004] Banerjee, S. and Goswami, R. (2013) GST profile expression study in some selected plants: in silico approach. Mol. Cell. Biochem. 380, 283–300. 10.1007/s11010-010-0384-y 20135200

[pbi12519-bib-0005] Barazesh, S. and McSteen, P. (2008) Barren inflorescence1 functions in organogenesis during vegetative and inflorescence development in maize. Genetics, 179, 389–401. 18493061 10.1534/genetics.107.084079 PMC2390617

[pbi12519-bib-0006] Berke, T.G. and Rocheford, T.R. (1999) Quantitative trait loci for tassel traits in maize. Crop Sci. 39, 1439–1443.

[pbi12519-bib-0502] Blatt, M. , Johansson, I. , Paneque, M. , Pratelli, R. , Campanoni, P. , Sokolovski, S. and Honsbein, A. (2008) SNAREs at the traffic junction with signalling, transport and nutrition. Comp Biochem Phys A 150, S141–S141.

[pbi12519-bib-0007] Boerjan, W. , Ralph, J. and Baucher, M. (2003) Lignin biosynthesis. Annu. Rev. Plant Biol. 54, 519–546. 14503002 10.1146/annurev.arplant.54.031902.134938

[pbi12519-bib-0008] Bogoeva, V.P. , Radeva, M.A. , Atanasova, L.Y. , Stoitsova, S.R. and Boteva, R.N. (2004) Fluorescence analysis of hormone binding activities of wheat germ agglutinin. Biochim. Biophys. Acta, 1698, 213–218. 15134654 10.1016/j.bbapap.2003.12.002

[pbi12519-bib-0009] Bolduc, N. , Yilmaz, A. , Mejia‐Guerra, M.K. , Morohashi, K. , O'Connor, D. , Grotewold, E. and Hake, S. (2012) Unraveling the *KNOTTED1* regulatory network in maize meristems. Genes Dev. 26, 1685–1690. 22855831 10.1101/gad.193433.112 PMC3418586

[pbi12519-bib-0010] Bomblies, K. , Wang, R.L. , Ambrose, B.A. , Schmidt, R.J. , Meeley, R.B. and Doebley, J. (2003) Duplicate FLORICAULA/LEAFY homologs zfl1 and zfl2 control inflorescence architecture and flower patterning in maize. Development, 130, 2385–2395. 12702653 10.1242/dev.00457

[pbi12519-bib-0011] Bommert, P. , Lunde, C. , Nardmann, J. , Vollbrecht, E. , Running, M. , Jackson, D. , Hake, S. *et al*. (2005) thick tassel dwarf1 encodes a putative maize ortholog of the Arabidopsis CLAVATA1 leucine‐rich repeat receptor‐like kinase. Development, 132, 1235–1245. 15716347 10.1242/dev.01671

[pbi12519-bib-0012] Brewbaker, J.L. (2015) Diversity and genetics of tassel branch numbers in maize. Crop Sci. 55, 65–78.

[pbi12519-bib-0013] Brown, P.J. , Upadyayula, N. , Mahone, G.S. , Tian, F. , Bradbury, P.J. , Myles, S. , Holland, J.B. *et al*. (2011) Distinct genetic architectures for male and female inflorescence traits of maize. PLoS Genet. 7, e1002383. 22125498 10.1371/journal.pgen.1002383 PMC3219606

[pbi12519-bib-0014] Buckler, E.S. , Holland, J.B. , Bradbury, P.J. , Acharya, C.B. , Brown, P.J. , Browne, C. , Ersoz, E. *et al*. (2009) The genetic architecture of maize flowering time. Science, 325, 714–718. 19661422 10.1126/science.1174276

[pbi12519-bib-0015] Chen, Z. , Wang, B. , Dong, X. , Liu, H. , Ren, L. , Chen, J. , Hauck, A. *et al*. (2014) An ultra‐high density bin‐map for rapid QTL mapping for tassel and ear architecture in a large F(2) maize population. BMC Genom. 15, 433. 10.1186/1471-2164-15-433 PMC4059873 24898122

[pbi12519-bib-0016] Chuck, G. , Muszynski, M. , Kellogg, E. , Hake, S. and Schmidt, R.J. (2002) The control of spikelet meristem identity by the branched silkless1 gene in maize. Science, 298, 1238–1241. 12424380 10.1126/science.1076920

[pbi12519-bib-0017] Chuck, G. , Meeley, R. , Irish, E. , Sakai, H. and Hake, S. (2007) The maize tasselseed4 microRNA controls sex determination and meristem cell fate by targeting Tasselseed6/indeterminate spikelet1. Nat. Genet. 39, 1517–1521. 18026103 10.1038/ng.2007.20

[pbi12519-bib-0018] Chuck, G. , Whipple, C. , Jackson, D. and Hake, S. (2010) The maize SBP‐box transcription factor encoded by tasselsheath4 regulates bract development and the establishment of meristem boundaries. Development, 137, 1243–1250. 20223762 10.1242/dev.048348

[pbi12519-bib-0019] Chuck, G.S. , Brown, P.J. , Meeley, R. and Hake, S. (2014) Maize SBP‐box transcription factors unbranched2 and unbranched3 affect yield traits by regulating the rate of lateral primordia initiation. Proc. Natl Acad. Sci. USA, 111, 18775–18780. 25512525 10.1073/pnas.1407401112 PMC4284592

[pbi12519-bib-0020] Colasanti, J. , Yuan, Z. and Sundaresan, V. (1998) The indeterminate gene encodes a zinc finger protein and regulates a leaf‐generated signal required for the transition to flowering in maize. Cell, 93, 593–603. 9604934 10.1016/s0092-8674(00)81188-5

[pbi12519-bib-0021] Cook, J.P. , McMullen, M.D. , Holland, J.B. , Tian, F. , Bradbury, P. , Ross‐Ibarra, J. , Buckler, E.S. *et al*. (2012) Genetic architecture of maize kernel composition in the nested association mapping and inbred association panels. Plant Physiol. 158, 824–834. 22135431 10.1104/pp.111.185033 PMC3271770

[pbi12519-bib-0022] Crue, R. and Wasson, C. (1996) Genetic analysis of tassel size and leaf senescence and their relationship with yield in two tropical low lands maize populations. Crop Sci. 4, 275–281.

[pbi12519-bib-0023] De Hoff, P.L. , Brill, L.M. and Hirsch, A.M. (2009) Plant lectins: the ties that bind in root symbiosis and plant defense. Mol. Genet. Genomics, 282, 1–15. 19488786 10.1007/s00438-009-0460-8 PMC2695554

[pbi12519-bib-0024] Dubots, E. , Audry, M. , Yamaryo, Y. , Bastien, O. , Ohta, H. , Breton, C. , Marechal, E. *et al*. (2010) Activation of the chloroplast monogalactosyldiacylglycerol synthase MGD1 by phosphatidic acid and phosphatidylglycerol. J. Biol. Chem. 285, 6003–6011. 20023301 10.1074/jbc.M109.071928 PMC2825394

[pbi12519-bib-0025] Duvick, D.N. and Cassman, K.G. (1999) Post‐green revolution trends in yield potential of temperate maize in the north‐central United States. Crop Sci. 39, 1622–1630.

[pbi12519-bib-0501] Elshire, R.J. , Glaubitz, J.C. , Sun, Q. , Poland, J.A. , Kawamoto, K. , Buckler, E.S. and Mitchell, S.E. (2011) A robust, simple genotyping‐by‐sequencing (GBS) approach for high diversity species. PLoS One 6, e19379. 21573248 10.1371/journal.pone.0019379 PMC3087801

[pbi12519-bib-0026] Eveland, A.L. , Goldshmidt, A. , Pautler, M. , Morohashi, K. , Liseron‐Monfils, C. , Lewis, M.W. , Kumari, S. *et al*. (2014) Regulatory modules controlling maize inflorescence architecture. Genome Res. 24, 431–443. 24307553 10.1101/gr.166397.113 PMC3941108

[pbi12519-bib-0027] Fornale, S. , Sonbol, F.M. , Maes, T. , Capellades, M. , Puigdomenech, P. , Rigau, J. and Caparros‐Ruiz, D. (2006) Down‐regulation of the maize and Arabidopsis thaliana caffeic acid O‐methyl‐transferase genes by two new maize R2R3‐MYB transcription factors. Plant Mol. Biol. 62, 809–823. 16941210 10.1007/s11103-006-9058-2

[pbi12519-bib-0028] Hallauer, A.R. and Miranda, J.B. (1988) Quantitative Genetics in Maize Breeding. Ames, IA: Iowa State University, 283.

[pbi12519-bib-0029] van Heerwaarden, J. , Hufford, M.B. and Ross‐Ibarra, J. (2012) Historical genomics of North American maize. Proc. Natl Acad. Sci. USA, 109, 12420–12425. 22802642 10.1073/pnas.1209275109 PMC3412004

[pbi12519-bib-0030] Hultquist, J.F. and Dorweiler, J.E. (2008) Feminized tassels of maize mop1 and ts1 mutants exhibit altered levels of miR156 and specific SBP‐box genes. Planta, 229, 99–113. 18800226 10.1007/s00425-008-0813-2

[pbi12519-bib-0031] Kindiger, B. , Blakey, C.A. and Dewald, C. (1995) Sex reversal in Maize X Tripsacum hybrids ‐ allelic non‐complementation of Ts2 And Gsf1. Maydica, 40, 187–190.

[pbi12519-bib-0032] Kriechbaumer, V. , Weigang, L. , Fiesselmann, A. , Letzel, T. , Frey, M. , Gierl, A. and Glawischnig, E. (2008) Characterisation of the tryptophan synthase alpha subunit in maize. BMC Plant Biol. 8, 44. 18430213 10.1186/1471-2229-8-44 PMC2395261

[pbi12519-bib-0033] Kump, K.L. , Bradbury, P.J. , Wisser, R.J. , Buckler, E.S. , Belcher, A.R. , Oropeza‐Rosas, M.A. , Zwonitzer, J.C. *et al*. (2011) Genome‐wide association study of quantitative resistance to southern leaf blight in the maize nested association mapping population. Nat. Genet. 43, 163–168. 21217757 10.1038/ng.747

[pbi12519-bib-0034] Li, H. , Ye, G. and Wang, J. (2007) A modified algorithm for the improvement of composite interval mapping. Genetics, 175, 361–374. 17110476 10.1534/genetics.106.066811 PMC1775001

[pbi12519-bib-0035] Li, C.H. , Li, Y.X. , Bradbury, P.J. , W, X. , Shi, Y.S. , Song, Y.C. , Zhang, D.F. *et al*. (2015) Construction of high‐quality recombination maps with low‐coverage genomic sequencing for joint linkage analysis in Maize. BMC Biol., 13, 78. 26390990 10.1186/s12915-015-0187-4 PMC4578237

[pbi12519-bib-0503] Lipka, A.E. , Tian, F. , Wang, Q. , Peiffer, J. , Li, J. , Bradbury, P.J. , Gore, M.A. *et al*. (2012) GAPIT: genome association and prediction integrated tool. Bioinformatics 28, 2397–2399. 22796960 10.1093/bioinformatics/bts444

[pbi12519-bib-0036] McMullen, M.D. , Kresovich, S. , Villeda, H.S. , Bradbury, P. , Li, H. , Sun, Q. , Flint‐Garcia, S. *et al*. (2009) Genetic properties of the maize nested association mapping population. Science, 325, 737–740. 19661427 10.1126/science.1174320

[pbi12519-bib-0037] McSteen, P. and Hake, S. (2001) barren inflorescence2 regulates axillary meristem development in the maize inflorescence. Development, 128, 2881–2891. 11532912 10.1242/dev.128.15.2881

[pbi12519-bib-0038] Mickelson, S.M. , Stuber, C.S. , Senior, L. and Kaeppler, S.M. (2002) Quantitative trait loci controlling leaf and tassel traits in a B73 x MO17 population of maize. Crop Sci. 42, 1902–1909.

[pbi12519-bib-0504] Mir, C. , Zerjal, T. , Combes, V. , Dumas, F. , Madur, D. , Bedoya, C. , Dreisigacker, S. *et al*. (2013) Out of America: tracing the genetic footprints of the global diffusion of maize. Theor Appl Genet 126, 2671–2682. 23921956 10.1007/s00122-013-2164-z

[pbi12519-bib-0039] Miyoshi, K. , Ahn, B.O. , Kawakatsu, T. , Ito, Y. , Itoh, J.I. , Nagato, Y. and Kurata, N. (2004) *PLASTOCHRON1*, a timekeeper of leaf initiation in rice, encodes cytochrome P450. Proc. Natl Acad. Sci. USA, 101, 875–880. 14711998 10.1073/pnas.2636936100 PMC321774

[pbi12519-bib-0040] Nishii, A. , Takemura, M. , Fujita, H. , Shikata, M. , Yokota, A. and Kohchi, T. (2000) Characterization of a novel gene encoding a putative single zinc‐finger protein, ZIM, expressed during the reproductive phase in *Arabidopsis thaliana* . Biosci. Biotechnol. Biochem. 64, 1402–1409. 10945256 10.1271/bbb.64.1402

[pbi12519-bib-0041] Peiffer, J.A. , Romay, M.C. , Gore, M.A. , Flint‐Garcia, S.A. , Zhang, Z. , Millard, M.J. , Gardner, C.A. *et al*. (2014) The genetic architecture of maize height. Genetics, 196, 1337–1356. 24514905 10.1534/genetics.113.159152 PMC3982682

[pbi12519-bib-0042] Petroni, K. and Tonelli, C. (2011) Recent advances on the regulation of anthocyanin synthesis in reproductive organs. Plant Sci. 181, 219–229. 21763532 10.1016/j.plantsci.2011.05.009

[pbi12519-bib-0043] Piperno, D.R. and Flannery, K.V. (2001) The earliest archaeological maize (*Zea mays* L.) from highland Mexico: new accelerator mass spectrometry dates and their implications. Proc. Natl Acad. Sci. USA, 98, 2101–2103. 11172082 10.1073/pnas.98.4.2101 PMC29388

[pbi12519-bib-0044] Preston, J.C. , Wang, H. , Kursel, L. , Doebley, J. and Kellogg, E.A. (2012) The role of teosinte glume architecture (tga1) in coordinated regulation and evolution of grass glumes and inflorescence axes. New Phytol. 193, 204–215. 21954998 10.1111/j.1469-8137.2011.03908.x

[pbi12519-bib-0045] Satoh‐Nagasawa, N. , Nagasawa, N. , Malcomber, S. , Sakai, H. and Jackson, D. (2006) A trehalose metabolic enzyme controls inflorescence architecture in maize. Nature, 441, 227–230. 16688177 10.1038/nature04725

[pbi12519-bib-0046] Schnee, C. , Kollner, T.G. , Gershenzon, J. and Degenhardt, J. (2002) The maize gene terpene synthase 1 encodes a sesquiterpene synthase catalyzing the formation of (E)‐beta‐farnesene, (E)‐nerolidol, and (E, E)‐farnesol after herbivore damage. Plant Physiol. 130, 2049–2060. 12481088 10.1104/pp.008326 PMC166716

[pbi12519-bib-0047] Taguchi‐Shiobara, F. , Yuan, Z. , Hake, S. and Jackson, D. (2001) The fasciated ear2 gene encodes a leucine‐rich repeat receptor‐like protein that regulates shoot meristem proliferation in maize. Genes Dev. 15, 2755–2766. 11641280 10.1101/gad.208501 PMC312812

[pbi12519-bib-0048] Tanaka, W. , Pautler, M. , Jackson, D. and Hirano, H.Y. (2013) Grass meristems II: inflorescence architecture, flower development and meristem fate. Plant Cell Physiol. 54, 313–324. 23378448 10.1093/pcp/pct016

[pbi12519-bib-0049] Tian, F. , Bradbury, P.J. , Brown, P.J. , Hung, H. , Sun, Q. , Flint‐Garcia, S. , Rocheford, T.R. *et al*. (2011) Genome‐wide association study of leaf architecture in the maize nested association mapping population. Nat. Genet. 43, 159–162. 21217756 10.1038/ng.746

[pbi12519-bib-0050] Tominaga‐Wada, R. , Iwata, M. , Nukumizu, Y. , Sano, R. and Wada, T. (2012) A full‐length R‐like basic‐helix‐loop‐helix transcription factor is required for anthocyanin upregulation whereas the N‐terminal region regulates epidermal hair formation. Plant Sci. 183, 115–122. 22195584 10.1016/j.plantsci.2011.11.010

[pbi12519-bib-0051] Upadyayula, N. , da Silva, H.S. , Bohn, M.O. and Rocheford, T.R. (2006) Genetic and QTL analysis of maize tassel and ear inflorescence architecture. Theor. Appl. Genet. 112, 592–606. 16395569 10.1007/s00122-005-0133-x

[pbi12519-bib-0052] Vedovato, M. , Rossi, V. , Dacks, J.B. and Filippini, F. (2009) Comparative analysis of plant genomes allows the definition of the “Phytolongins”: a novel non‐SNARE longin domain protein family. BMC Genom., 10, 1–13. 10.1186/1471-2164-10-510 PMC2779197 19889231

[pbi12519-bib-0053] Vollbrecht, E. , Springer, P.S. , Goh, L. , Buckler, E.S. and Martienssen, R. (2005) Architecture of floral branch systems in maize and related grasses. Nature, 436, 1119–1126. 16041362 10.1038/nature03892

[pbi12519-bib-0054] Walsh, J. , Waters, C.A. and Freeling, M. (1998) The maize gene liguleless2 encodes a basic leucine zipper protein involved in the establishment of the leaf blade‐sheath boundary. Gene Dev. 12, 208–218. 9490265 10.1101/gad.12.2.208 PMC316436

[pbi12519-bib-0055] Wang, R. , Yu, Y. , Zhao, J. , Shi, Y. , Song, Y. , Wang, T. and Li, Y. (2008) Population structure and linkage disequilibrium of a mini core set of maize inbred lines in China. Theor. Appl. Genet. 117, 1141–1153. 18696041 10.1007/s00122-008-0852-x

[pbi12519-bib-0056] Wang, T.Y. , Ma, X.L. , Li, Y. , Bai, D.P. , Liu, C. , Liu, Z.Z. , Tan, X.J. *et al*. (2011) Changes in yield and yield components of single‐cross maize hybrids released in China between 1964 and 2001. Crop Sci. 51, 512–525.

[pbi12519-bib-0057] Wenefrida, I. , Utomo, H.S. and Linscombe, S.D. (2013) Mutational breeding and genetic engineering in the development of high grain protein content. J. Agric. Food Chem. 61, 11702–11710. 23869957 10.1021/jf4016812

[pbi12519-bib-0058] White, D.W.R. (2006) PEAPOD regulates lamina size and curvature in Arabidopsis. Proc. Natl Acad. Sci. USA, 103, 13238–13243. 16916932 10.1073/pnas.0604349103 PMC1550771

[pbi12519-bib-0059] Wu, X. , Li, Y. , Shi, Y. , Song, Y. , Wang, T. , Huang, Y. and Li, Y. (2014) Fine genetic characterization of elite maize germplasm using high‐throughput SNP genotyping. Theor. Appl. Genet. 127, 621–631. 24343198 10.1007/s00122-013-2246-y

[pbi12519-bib-0060] Yan, J. , Shah, T. , Warburton, M.L. , Buckler, E.S. , McMullen, M.D. and Crouch, J. (2009) Genetic characterization and linkage disequilibrium estimation of a global maize collection using SNP markers. PLoS ONE, 4, e8451. 20041112 10.1371/journal.pone.0008451 PMC2795174

[pbi12519-bib-0061] Yang, N. , Lu, Y.L. , Yang, X.H. , Huang, J. , Zhou, Y. , Ali, F. , Wen, W.W. *et al*. (2014) Genome wide association studies using a new nonparametric model reveal the genetic architecture of 17 agronomic traits in an enlarged maize association panel. PLoS Genet. 10, e1004573. 25211220 10.1371/journal.pgen.1004573 PMC4161304

[pbi12519-bib-0062] Zhang, Z.W. , Ersoz, E. , Lai, C.Q. , Todhunter, R.J. , Tiwari, H.K. , Gore, M.A. , Bradbury, P.J. *et al*. (2010) Mixed linear model approach adapted for genome‐wide association studies. Nat. Genet. 42, 355–U118. 20208535 10.1038/ng.546 PMC2931336

