# Supplementary figures and images for: Joint‐linkage mapping and GWAS reveal extensive genetic loci that regulate male inflorescence size in maize
Source: Plant Biotechnol J. 2016 Jan 23;14(7):1551–62. doi: 10.1111/pbi.12519 (PMC5066742; doi:10.1111/pbi.12519)

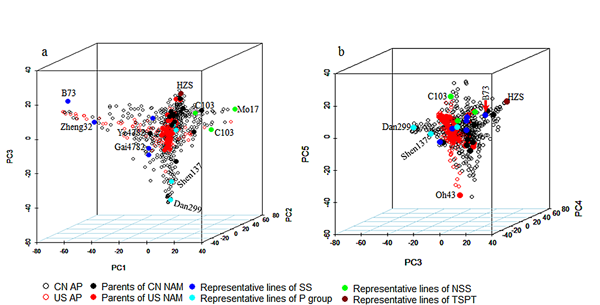

Supplement: Supplementary file 1 — Figure S1 Scatter plot of the first three principal components. [file PBI-14-1551-s004.tif]

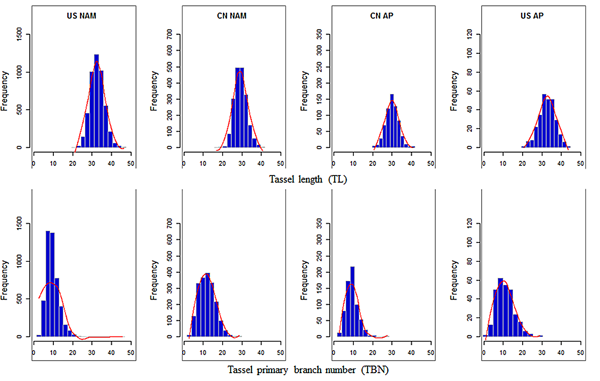

Supplement: Supplementary file 2 — Figure S2 Phenotypic variations of tassel‐related traits. [file PBI-14-1551-s003.tif]

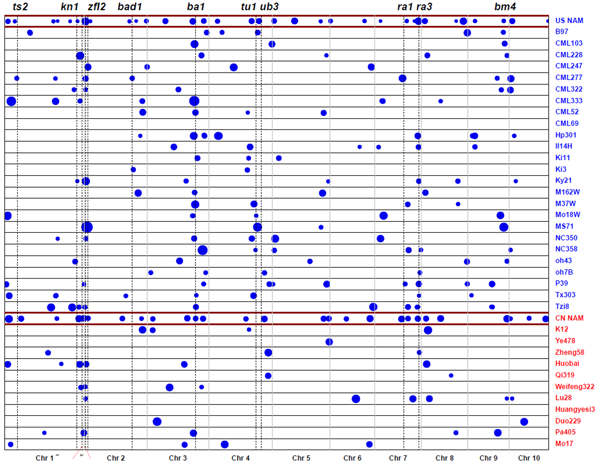

Supplement: Supplementary file 3 — Figure S3 QTL associated with tassel primary branch number (TBN) across the 36 RILs families. [file PBI-14-1551-s002.tif]

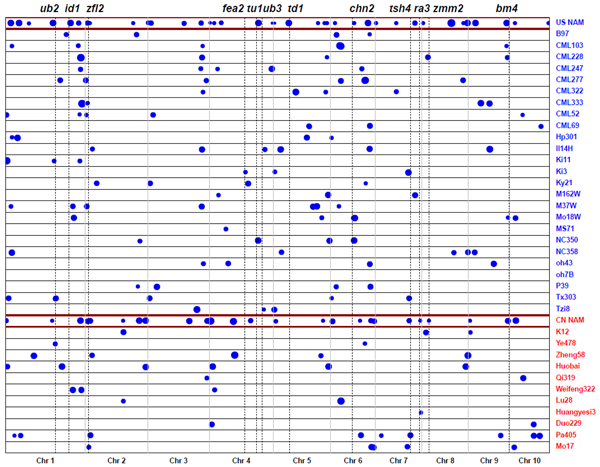

Supplement: Supplementary file 4 — Figure S4 QTL associated with tassel length (TL) across 36 RILs families. [file PBI-14-1551-s005.tif]
